# Overconfidence in Understanding of How Electronic Gaming Machines Work Is Related to Positive Attitudes

**DOI:** 10.3389/fpsyg.2020.609731

**Published:** 2021-01-12

**Authors:** Kahlil S. Philander, Sally M. Gainsbury

**Affiliations:** ^1^School of Hospitality Business Management, Carson College of Business, Washington State University, Everett, WA, United States; ^2^Science Faculty, Brain and Mind Centre, School of Psychology, University of Sydney, Camperdown, NSW, Australia

**Keywords:** electronic gaming machines, skill, attitudes, illusions of control, erroneous beliefs, cognitive distortions, misunderstanding, gambling

## Abstract

Previous research has demonstrated that attitudes are a primary determinant of intention to gamble on electronic gaming machines (EGMs) consistent with the Theory of Reasoned Action. This paper aims to address how biases in judgment can contribute to attitudes and subsequently behavior, including maladaptive problematic gambling behavior. We take a novel approach by viewing overconfidence in one’s understanding of how outcomes are determined on EGMs as an indication of cognitive distortions. The novelty of this paper is further increased as we compare attitudes to existing EGMs with novel EGMs which include a skill component, referred to as skill-based gaming machines (SGMs), which enables a better controlled comparison between actual and perceived skill. In Study 1, 232 US-based participants were recruited online who were shown various slot machines and SGMs and asked a series of questions about perceived skill and chance in determining outcomes to assess their understanding, then were asked their confidence in their understanding, attitudes toward the machines and they completed the Problem Gambling Severity Index. In Study 2, 246 Australian participants were recruited through community and university student samples; they attended a laboratory where they were randomly allocated to play a real EGM or SGM without money and completed the same measures as in Study 1. In Study 2, participants were randomly told that the outcomes on the machine they would play were determined entirely by chance, skill, or a mixture of both. In both studies, our findings suggest that there are more extreme values in overconfidence in how EGMs work, whereas individuals are more similar in their confidence in understanding SGMs. We also find a relationship between overconfidence in EGM understanding and positive attitudes toward EGMs, but no such relationship with SGMs. There was no impact from controlling for demographics, problem gambling severity, or labeling of machines on these relationships.

## Introduction

Gambling is a popular activity internationally, with past-year participation rates varying from 58 to 83% (Dowling et al., 2016; Castrén et al., 2018; Salonen et al., 2018; Volberg et al., 2018). Research often focuses on what contributes to excessive gambling, but there is a significant research gap to inform why people gamble, including uptake of novel gambling activities. There is evidence to support the Theory of Reasoned Action (TRA, Fishbein and Ajzen, 1975) to explain intentions to gamble, including on electronic gaming machines (EGMs), i.e., slots, pokies, fixed odds betting terminals (Thrasher et al., 2011; Lee, 2013; Shin and Montalto, 2015; Gainsbury et al., 2019a). According to the TRA, intent to gamble is predicated on both positive attitudes toward the activity and perceived social norms. However, there is an absence of research to understand what contributes to positive attitudes toward various gambling activities.

Despite policies mandating gambling product information disclosure, erroneous beliefs about gambling and erroneous understandings of how outcomes are determined are widely held by gambling consumers (Goodie et al., 2019). Due to the potential for substantial money spending in the case of gambling, erroneous beliefs about gambling may lead to serious harms (Goodie et al., 2019).

One of the empirical challenges in understanding the link between electronic gaming machines (EGMs), erroneous beliefs, and attitudes toward gambling, relates to the role of user actions in generating the random outcomes. A new gambling product has been developed, similar to EGMs but with notable differences in how outcomes are determined through the inclusion of skill-based outcomes, which are referred to here as skill gaming machines (SGMs). This development provides an opportunity to examine the relationship between attitudes toward a gambling product and erroneous understanding of how outcomes are determined, in the form of overconfidence in understanding of the machines. This paper considers the extent to which consumers understand the newer SGMs in comparison to EGMs, the impact of labels explaining how outcomes are determined, and the role of actual and subjective understanding of the differing machines on positive product attitudes.

## Literature Review

Gambling-related erroneous beliefs tend to be based on a misunderstanding of how outcomes are determined and can impact gambling, and can contribute to the development of gambling problems (Goodie et al., 2019). Overestimation of control over outcomes strengthens individuals’ win expectations, leading players to place higher bets and persist in betting (Kwak, 2016). Distorted cognitions can moderate the relationship between risky gambling practices and spending (Miller and Currie, 2008) and erroneous beliefs are an independent predictor of problem gambling severity (MacKay and Hodgins, 2012). Illusions of control and other erroneous beliefs, such as a belief in luck, may be more common in relation to gambling activities that include a skill element or structural characteristics that encourage the perception of skill. Individuals who prefer gambling activities that contain elements of skill have a greater illusion of control over outcomes (Myrseth et al., 2010). A study of online poker players found those who did not overestimate their skill were more successful at avoiding developing gambling problems (Griffiths et al., 2010), suggesting that avoiding cognitive distortions may be protective against problematic gambling. Given the potential influence of erroneous beliefs on behavior, it is important to identify the relationship between these and attitudes toward gambling activities.

One limitation of erroneous belief measures is that they generally attempt to estimate fallacies across multiple activities or the entire set of gambling activities (Goodie and Fortune, 2013; Goodie et al., 2019). These measures provide limited insight as to how attitudes toward an individual game or game type are shaped, which is an important consideration for policy and lower-risk game design. While there are likely to be global effects of cognitive distortions that impact individuals behavior across all gambling variants, we contend that attitudes toward any particular game can be explained, in part, by the degree of overconfidence that the individual has toward that game, in their ability to control outcomes. There is minimal research on the role of overconfidence in how outcomes are determined in the gambling field. One study found that individuals with gambling problems are more likely to be overconfident in tasks involving skills and were more likely to wager that they were correct in their performance of skilled activities in a simulated gambling task compared to individuals without gambling problems (Goodie, 2005). In this paper we take a novel approach in viewing overconfidence as a potential cognitive distortion; if an individual has a poor understanding, but their subjective assessment of their understanding matches that uncertainty, then there are no distortions. We expect individuals could accurately calibrate their risk taking, based on their matched understanding and subjective uncertainty.

EGMs are of central interest to gambling regulators and researchers, given their propensity to be related to gambling problems (Delfabbro et al., 2020b). EGMs are randomly determined, however, the products often include redundant features that reinforce an illusion of control, such as bonus rounds or stop buttons, which appear to tie user actions to outcomes (Harrigan et al., 2014; Gainsbury et al., 2018). Until recently, there were no suitable control devices that facilitated a comparison to the false appearance of skill in EGMs. However, SGMs have been developed which incorporate skill elements into the randomly determined payout schedules of EGMs (for a review please see Delfabbro et al., 2020a; Pickering et al., 2020). These machines allow for player skill to impact the long-run house advantage such that all players have the possibility of winning, including jackpots, but players with higher levels of skill increase their likelihood of winning small to medium monetary payouts.

This is similar to other gambling activities, for example casino-based poker or blackjack, whereby greater skill provides the player with an advantage such that skill players are more likely to win than unskilled players, but the house retains an advantage to ensure that most players will lose in the long-run. Unlike poker and blackjack, the skill mechanics within SGMs are modeled on video and mobile games (which are not classified as gambling as they do not provide monetary payouts) and may include pattern matching, fighter, or sports-based player actions. SGMs are often physically different from EGMs and include touchscreen or video-game-style controllers and considerable interactivity and decision-making. Several, predominantly US, jurisdictions have enacted legislation permitting SGMs and other jurisdictions are permitting SGMs within existing EGM regulation frameworks. However, policy makers and stakeholders have expressed concerns regarding the extent to which SGMs may exacerbate gambling harms (Hoskins and Hoskins, 2019). One concern is that consumers may not understand the extent to which skill impacts SGM outcomes in relation to chance and that subsequently, consumers will develop erroneous beliefs which may increase risky gambling including excessive play and chasing losses and lead to harms.

There is limited empirical research on SGMs related to their impact or the extent to which players understand these devices. The current paper builds on published results by the authors, which found that after reading a description of SGMs and viewing a brief video of examples, participants recruited from a US-based online sample understood that SGMs involved more skill than EGMs, but they were not confident that they understood how the machines worked (Gainsbury et al., 2020). Further, compared to participants with no prior SGM experience, participants who had experience gambling on SGMs had a poorer understanding of how outcomes were determined for EGMs but not SGMs. They also had higher rates of gambling-related cognitive distortions in general and higher problem gambling severity scores. This indicates that experience playing SGMs did not significantly enhance understanding of SGMs and that individuals already involved in gambling and with existing cognitive distortions and gambling problems are likely to play these new products. Similar results were found in a related study of casino patrons who played a SGM (Gainsbury et al., 2019b). Following play, participants did not have a good understanding of how outcomes were determined for SGMs, and this did not differ based on prior use of SGMs in casinos. Participants with greater gambling-related erroneous beliefs, including illusions of control, and gambling problems were more likely to have played and report interest in playing SGMs.

Many harm prevention strategies involve the provision of information intended to educate consumers about game play for example through messages on products, or signs and brochures in venues. These strategies persist despite repeated research showing that knowledge of gambling odds and information about gambling is unlikely to impact gambling behaviors (Monaghan and Blaszczynski, 2009; Parke et al., 2014; Ginley et al., 2017). A common legislative requirement across jurisdictions that were early adopters in permitting skill-based gambling, is clear labeling of SGMs as containing skill-elements (Larche et al., 2016). However, given the tendency for EGM play information to fail to influence behavior, it is possible that similar signage for SGMs is ineffective in impacting cognitions.

The TRA is a well-established social cognitive model which posits that behavior is determined by an individual’s intent to perform that behavior, which is in turn predicted by attitudes toward the behavior and perceived social norms toward the behavior (i.e., a perception of how others perceive the behavior). The TRA has been previously applied to gambling with evidence supporting this theoretical framework (Cummings and Corney, 1987; Moore and Ohtsuka, 1997; Thrasher et al., 2011; Lee, 2013; Shin and Montalto, 2015). Preliminary evidence supports the TRA as an explanatory model for understanding intent to gamble on EGMs and SGMs which found more positive attitudes and stronger subjective norms predicted a stronger intention to gamble, and this finding was stronger for SGMs than EGMs (Gainsbury et al., 2019a). The current investigation uses the data from this study and aims to extend the published results by examining an antecedent of attitudes (Study 1) and addresses the MTurk study data limitations with a subsequent lab-based study (Study 2). As the TRA is a relevant conceptual model to understand gambling behavioral intention and subsequent action it is important to identify what factors influence the sub-components, including personal attitudes toward specific gambling activities and products, given the role of these in determining behavior.

### Current Study

The current research aimed to examine the role of overconfidence in EGM and SGM game understanding, testing whether overconfidence predicted attitudes toward the game types, and testing whether simple labels providing information about how outcomes are determined will impact this relationship. Study One presents further analyses from the previously mentioned US-online dataset (Gainsbury et al., 2020) and Study Two presents analyses from a subsequent Australian lab-based study, which aimed to overcome some of the limitations in Study One. Specifically, compared to Study One, Study Two recruited a broader sample from a different geographic location with no previous exposure to SGMs and provided participants with a notice of how outcomes were determined. Study Two randomly allocated participants to either play and respond regarding SGMs or EGMs, unlike Study One, which asked about both types of machines without random allocation. The outcomes of this research make an important Contribution To The Field by advancing understanding of whether cognitive distortions measured by overconfidence in an individual’s understanding of how outcomes are determined, predicts attitudes toward EGMs and newly introduced SGMs, which introduce an element of skill to EGMs. The implications of this research are relevant for policy makers in determining whether interventions which enhance accuracy of understanding how outcomes are determined for existing and novel gambling activities are an appropriate harm minimization measure.

We hypothesize that game understanding is important and systematically misunderstood by EGM players. In SGMs, we expect similar distributions of game understanding but given that the skill component is more obvious, we expect individuals to project their subjective skill level more correctly.

## Study 1

Study 1 was a secondary analysis of an observational study administered through an online survey. The study included several questions about the nature of skill and chance in SGMs and EGMs, along with self-reported measures. Ethics clearance for Study 1 was provided by University of Sydney Human Research Ethics Committee 2017-890. The study was not pre-registered, but the measures used and data analyzed are freely available at: https://osf.io/utf9z/.

### Materials and Methods

#### Participants

A sample was recruited in November 2017 using Amazon Mechanical Turk (MTurk), an online platform for tasks. Participants were restricted to legal gambling age (21 years of age or older) individuals with an MTurk approval rating of at least 95% (Goodman et al., 2013), who speak English, and who live in North America. Respondents must also had resided in or visited a jurisdiction that contained the games shown in the study. A total of 232 respondents were recruited and 48 were removed from analysis due to failing at least one of two attention checks or not completing the survey.

#### Procedure

Participants were shown brief videos^1^ within the survey depicting various SGMs and EGMs and were asked a series of questions about perceived skill and chance in gambling and non-gambling games.

#### Measures

##### Game Understanding

Respondents were asked similar, but separate, game understanding questions about EGMs and SGMs. Consistent with regional terminology, EGMs were described as “slot machines.” The questions were then scored differently to reflect accuracy of the responses (measured game understanding). Items were coded on a five-point Likert scale (1, *strongly disagree*; 5, *strongly agree*) and a summative accuracy score from 4 to 20 was computed. The questions were:

• *A player of greater skill is more likely to win money on (slot machines/skill-based gambling machines) over 1 h of play, compared to a player of lesser skill.* (Reverse scored for slot machines).
• Over the long term, all players will lose money on (slot machines/skill-based gambling machines).
• *The outcomes of (slot machines/skill-based gambling machines) are random no matter what a player does.* (Reverse scored for SGMs).
• *With practice a player can improve their outcomes on (slot machines/skill-based gambling machines) over time.* (Reverse scored for slot machines).

Across all individuals, the difference in average scores for SGM understanding (*M* = 15.27, *SD* = 2.22) and slot machine understanding (*M* = 16.04, *SD* = 3.43) was small but statistically significant using Welch’s unequal variances *t*-test, *t*(314) = −2.54, *p* = 0.01.

##### Self-Reported Game Understanding

Self-reported game understanding was coded on a five-point Likert scale (1, *strongly disagree*; 5, *strongly agree*). The question was:

• I understand how a player’s skill impacts the outcomes of (slot machines/skill-based gambling machines).

The difference in average scores for self-reported SGM understanding (*M* = 3.99, *SD* = 0.93) and self-reported slot understanding (*M* = 2.91, *SD* = 1.42) was over one unit on the scale and statistically significant using Welch’s unequal variances *t*-test, *t*(315) = 8.64, *p* < 0.001.

##### Game Attitudes

Three items assessed the appeal, excitement, and enjoyableness of (*slot machines/skill-based gambling machines*). These measures have previously been used to assess attitudes and were found to be predictive of future intent to gamble on machines (Gainsbury et al., 2019a). Items were assessed on a 5-point Likert scale (1, very unenjoyable; 5, very enjoyable). Higher scores indicate a more positive attitude. The items showed adequate consistency using Cronbach’s alpha (α) in slot machines, α = 0.92, and SGMs, α = 0.90.

##### Problem Gambling Severity Index

Respondents were asked questions from the problem gambling severity index (PGSI) (Ferris and Wynne, 2001), as gambling problems are potentially related to both attitudes and overconfidence. We score the index using categories suggested by Currie et al. (2013), as these are the points that have been found as valid for inference. The sample classified participants into one of four groups: non-problem/non-gambling (*n* = 83; 45.11%), low-risk gambling (*n* = 48; 26.09%), moderate-risk gambling (*n* = 13; 7.07%), and problem gambling (*n* = 40; 21.74%).

##### Demographic Variables

Respondents were asked to provide their age (*M* = 34.02, *SD* = 9.29), gender (32.07% female, 67.93% male), employment status (full-time = 78.26%, part-time = 9.24%, unemployed = 4.89%, other = 7.61%), and household income band (median band = USD 50,000–70,000).

### Analysis

To test for the relation between overconfidence and attitudes toward the electronic gaming machines, we regress measures of overconfidence onto the attitude factor variables in a series of ordinary least square models with sensitivity analysis to demonstrate the robustness of the results (Leamer, 1985). Our sensitivity analysis procedure includes first estimating a simple regression model, then estimating a second model that includes potentially confounding PGSI categories, and finally a fully specified model that adds demographic controls.

#### Overconfidence Measures

Measures of overconfidence were computed as the difference between standardized measured game understanding and standardized self-reported game understanding, for both game variants, respectively. That is,

$$O\,v\,e\,r\,c\,o\,n\,f\,i\,d\,e\,n\,t_{i}^{j}=(S\,e\,l\,f\,r\,e\,p\,o\,r\,t\,e\,d\,u\,n\,d\,e\,r\,s\,\tan d\,i\,n\,g_{i}^{j}-m\,e\,a\,s\,u\,r\,e\,d\,u\,n\,d\,e\,r\,s\,\tan d\,i\,n\,g_{i}^{j})$$

Where, the respondent is denoted by *“i”* and the activity (Slots or SGMs) is denoted by *“j.”* Intuitively, we measure whether respondents self-reported level of game understanding is near our measured level, with higher values denoting greater overconfidence and thus greater misunderstanding of the nature of the game. Prior to computing overconfidence, we standardize our measures of understanding with z-scores, to place them on similar scales.

Overconfidence can be viewed as an overestimation of understanding of how games work or an overestimation of control over outcomes^2^. As our measures of understanding do not distinguish between these concepts, we treat our overconfidence variable as a more general measure of these specific phenomena. Table 1 includes summary statistics of the attitude, overconfidence, and standardized understanding variables.

**TABLE 1:** Summary Statistics.

	Count	Mean	SD	Min	Max
Self-reported slot understanding (z-score)	184	0.000	1.000	−1.344	1.466
Self-reported SGM understanding (z-score)	184	0.000	1.000	−3.233	1.086
Measured slot understanding (z-score)	184	0.000	1.000	−2.049	1.154
Measured SGM understanding (z-score)	184	0.000	1.000	−3.729	2.132
Slot attitude factor	184	0.000	0.947	−2.524	1.200
SGM attitude factor	184	0.000	0.926	−3.860	1.006
Slot overconfidence	184	0.000	1.658	−2.498	3.225
SGM overconfidence	184	0.000	1.010	−3.834	3.462

**FIGURE 1:**
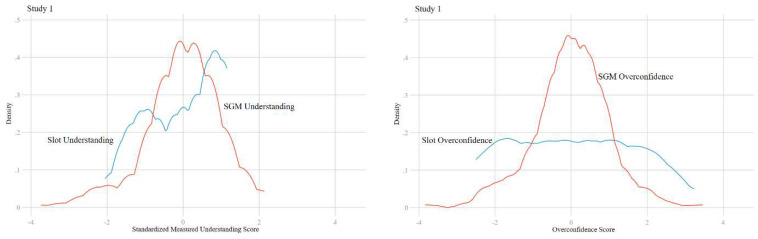
Kernel density plots of measured understanding and overconfidence scores by machine type for study 1. The distribution of overconfidence shows large fat-tail effects in slot machines but not SGMs.

Figure 1 illustrates the Kernel density plots of understanding and overconfidence variables. SGM understanding appears relatively normally distributed, but there is left-skewness in the distribution of slot machine understanding. This skewness contributes to a fat-tail in the distribution of overconfidence. A larger share of respondents have a high level of overconfidence about their understanding of slot machines, as compared to SGMs.

## Results

We found evidence suggesting that overconfidence is positively related to attitudes toward slots. As shown in Table 2, computed estimates suggest that each unit in the slot overconfidence scale is related to a 1.28–1.67 increase in the slot attitude factor scores, depending on the model specification. We do not find a significant relation of SGM overconfidence to SGM attitudes. These findings are robust to inclusion of control variables for PGSI categories, age categories, gender, employment status, and household income. We also find that individuals in the low and moderate PGSI categories have more positive attitudes toward slots and SGMs than gamblers in the non-problem category. The relationship is less robust for individuals in the PGSI problem category, where we do not find a significant effect in either of our fully specified models.

**TABLE 2:** Ordinary least squares regression of overconfidence variables onto attitude factor variables (online survey).

	Slots	Slots	Slots	SGMs	SGMs	SGMs
Slots-	0.167***	0.128*	0.132*			
Overconfidence	(0.041)	(0.053)	(0.059)			
SGM-				−0.019	0.018	0.034
Overconfidence				(0.088)	(0.089)	(0.091)
PGSI low		0.442*	0.387*		0.556***	0.524**
		(0.170)	(0.181)		(0.154)	(0.164)
PGSI moderate		0.866***	0.728***		0.765***	0.739**
		(0.186)	(0.201)		(0.189)	(0.223)
PGSI problem		0.407*	0.362		0.060	0.002
		(0.160)	(0.202)		(0.163)	(0.214)
Constant	0.000	−0.265*	−0.202	−0.000	−0.212	−0.228
	(0.067)	(0.113)	(0.223)	(0.068)	(0.127)	(0.236)
Age categories	No	No	Yes	No	No	Yes
Gender	No	No	Yes	No	No	Yes
Employment	No	No	Yes	No	No	Yes
Household income	No	No	Yes	No	No	Yes
*N*	184	184	184	184	184	184
*R*^2^	0.086	0.159	0.252	0.000	0.090	0.204

*p < 0.05, **p < 0.01, ***p < 0.001. Heteroskedasticity robust standard errors shown in brackets. PGSI measures are binary variables representing categories in the problem gambling severity index, with non-problem/non-gambling excluded as the base category.

## Study 2

Study 1 demonstrated a different relation between EGM and SGM overconfidence and player attitudes, but there may have been confounds from comparisons made by respondents, as they were asked to rate both machines simultaneously. It is also unclear if the media fully illustrated the user experience on these gaming machines. To address these deficiencies, Study 2 was a laboratory study, involving play by subjects on actual SGMs and EGMs in a demonstration mode. The study included several questions about the nature of skill and chance in SGMs and EGMs, but we address the potential confounding issues in Study 1 by randomly assigning subjects to either SGMs or EGMs. Ethics clearance for study 2 was provided by the University of Sydney Human Research Ethics Committee (2010-738).

Given the propensity for individuals to hold cognitive biases, our pre-registered hypothesis was that participants who played machines with skill labels will have a less accurate understanding of the outcomes of machines than those who played machines labeled as determined by chance or with no label at all. As there is minimal literature to guide expectations, our pre-registered exploratory analysis was to investigate the impact of framing on understanding of SGM/EGMs and irrational beliefs. Finally, we explored whether labeling impacted variables that may be indicative of gambling-related harm, including future intent to play, immersion, craving, perceptions of skill vs. chance and irrational beliefs. In addition, we conducted a non-pre-registered exploration of the impact of mis-labeling machines on erroneous beliefs and game understanding. The pre-registration details, measures, and data are available at https://osf.io/ba5n2/.

### Materials and Methods

#### Participants

The study aggregated subjects sampled from three target groups within the Australian population: (i) young adults (aged 18–39 years), since this population is a potential target audience for SGMs (Delfabbro et al., 2020a; Pickering et al., 2020) and have relatively high rates of gambling and gaming-related problems (Gainsbury et al., 2014; Welte et al., 2015); (ii) regular EGM users, since such individuals would likely encounter SGMs if they were made available in gambling venues; and (iii) community members, since it is important to understand the potential appeal and impact of SGMs on individuals who may not regularly attend licensed gambling venues but may be interested in skill-related games. The use of multiple sampling strategies improves our study validity, as convenience samples like college students may not generalize well when attitudinal variables are used (Hanel and Vione, 2016).

To participate, respondents had to be at least 18 years of age, an Australian resident, and fluent in English. Young adults were recruited via an online research participant recruitment platform hosted by the (University redacted). This platform allows students to sign up to participate in research studies as part of a voluntary research participation assessment component in exchange for course credit. Students outside of the research participation assessment scheme can also sign up to participate in studies and are offered a monetary reimbursement for their time. Regular EGM users were recruited by distributing 500 leaflets in a local gambling venue and by posting a recruitment notice in an e-newsletter distributed to club members. Additional participants who reported playing EGMs at least monthly were recruited through a recruitment agency. Community members were recruited through word-of-mouth and social media posts, and via a recruitment agency. A total of 133 student respondents and 113 community members were recruited in November/December 2019, for an aggregate sample of 246 individuals.

#### Procedures

Upon arrival, consenting participants completed *pre-test* questionnaires using tablet devices. Once all participants had completed the *pre-test* questionnaire, they were taken by a researcher to a room housing three EGMs and three SGMs. Up to six participants were included in each session. Machines were pre-loaded with credit. The researcher instructed each participant to sit at the specific gaming machine corresponding to the experimental condition to which they had been randomly assigned.

Each machine additionally had a labeling condition to which participants were randomly assigned. The appearance of a machine as having a skill component through design elements, such as a video game style controller, may have an impact on the individual’s thoughts and behaviors. To control for the effects of framing gaming machine outcomes as being influenced by skill or chance, the description of the machines provided to participants in the *pre-test* questionnaire differed across three categories: (i) “outcomes on the machine you are going to play are determined by a mix of skill and chance” (“skill label”); (ii) “outcomes on the machine you are going to play are determined completely by chance” (“chance label”); or (iii) no reference was made to the role of skill or chance (“no label”). We summarize the conditions in Table 3, and use these labeling conditions as additional explanatory variables.

**TABLE 3:** Experimental conditions.

	Framing label		
	
Machine type	Skill label	Chance label	No label
EGM	Condition 1	Condition 2	Condition 3
SGM	Condition 4	Condition 5	Condition 6

All machines were set to “demo mode” such that no real money was involved in playing the machines, however, the machines operated as they would if they were in a licensed venue. The EGM was a standard reel-based game which included bonus rounds in which participants were shown a deck of cards and could select “red/black” for the next card to be drawn to win an additional prize. The SGM had two play components, the “chance” component consisted of reel-spins as in regular EGMs and the “skill” component was a bonus feature in which participants entered a battle scene and used the video-game controller to fight monsters while acting as a Knight-style avatar^3^. No credits were bet in the skill gaming component, however, superior performance in this component may result in a “win” for participants and increase their credit total. Participants were instructed to play the machines for 20 min. Participants were then asked to complete the *post-test* questionnaire using tablet devices. Once all participants had completed the *post-test* questionnaire, the researcher provided a verbal debrief to ensure that participants understood the experimental protocol, and the role of skill and chance in determining outcomes in each machine type. Participants were able to ask any questions about their experience playing the machines. Participants were awarded course credit or offered a monetary reimbursement for their time.

#### Measures

A similar set of variables to those used in Study 1 appeared in the survey instrument from Study 2, including game understanding, self-reported game understanding, game attitudes, game intentions, and SGM/EGM overconfidence. Household income values were collected as Australian dollars, and age was collected as a non-categorical variable to allow for its use as a continuous regression input. We use two additional variable, “told skill,” which is a dummy variable equal to “1” if their machine had a skill label and “0” otherwise; and “told chance,” which is a dummy variable equal to “1” if their machine had a chance label and “0” otherwise. A total of 75 participants received the skill label and 93 received the chance label. The average age was 34.04 (*SD* = 17.32), gender (female = 56.91%, male = 42.68%, other = 0.41%), employment status (student = 39.43%, full-time = 24.39%, part-time = 22.36%, unemployed = 3.25%, other = 10.57%), and household income band (median band = AUD 65,000–77,999 per year).

Across all respondents, there were 105 non-gamblers/non-problem gamblers (41.50%), 87 low-risk gamblers (34.39%), 31 moderate-risk gamblers (12.25%), and 30 problem gamblers (11.86%). There were 140 females (56.91%), 105 males (42.68%), and 1 non-binary (0.41%) subjects. There were 115 respondents reporting as working in a paid job (46.75%), 97 as students (39.43%), and 34 in other employment circumstances (13.82%).

Between groups, the difference in average scores for SGM understanding (*M* = 12.40, *SD* = 2.92) and EGM understanding (*M* = 15.49, *SD* = 3.47) was significant, using Welch’s unequal variances *t*-test, *t*(237) = −7.55, *p*< 0.001. Self-reported SGM understanding (*M* = 3.10, *SD* = 1.23) was higher than self-reported EGM understanding (*M* = 2.43, *SD* = 1.34) using Welch’s unequal variances *t*-test, *t*(243) = 4.10, *p* < 0.001.

Table 4 summarizes non-categorical variables used in the study, including standardized versions of the understanding measures.

**TABLE 4:** Summary statistics.

	Count	Mean	*SD*	Min	Max
Self-reported EGM understanding (z-score)	122	0.000	1.000	−1.074	1.920
Self-reported SGM understanding (z-score)	124	0.000	1.000	−1.713	1.543
Measured EGM understanding (z-score)	122	0.000	1.000	−3.024	1.299
Measured SGM understanding (z-score)	124	0.000	1.000	−2.192	2.600
EGM overconfidence	122	0.000	1.787	−2.373	4.656
SGM overconfidence	124	0.000	1.107	−3.287	3.050
EGM attitudes factor	122	0.000	1.000	−2.241	1.697
SGM attitudes factor	124	0.000	1.000	−1.938	1.862
Age	246	34.044	17.324	18	75

**FIGURE 2:**
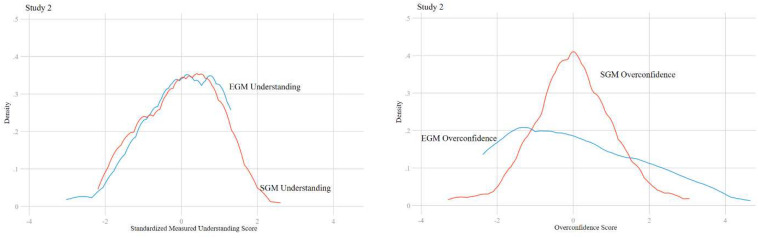
Kernel density plots of measured understanding and overconfidence scores by machine type for study 2. The distribution of overconfidence shows large fat-tail effects in EGMs but not SGMs.

Figure 2 illustrates the Kernel density plots of the understanding variables, and the overconfidence variables. We observe that SGM understanding appears relatively normally distributed, but there is left-skewness in the distribution of EGM understanding. This skewness contributes to a fat-tail in the distribution of overconfidence. A larger share of respondents have a high level of overconfidence about their understanding of EGMs, as compared to SGMs.

#### Analysis

To test for the relationship between overconfidence and attitudes toward the gambling machines, we regress overconfidence onto the factor variables. We then fit multivariate models to assess the robustness of the findings, which includes the mislabelled machine condition. Our sensitivity analysis procedure consolidates the PGSI and demographic control addition step used in Study 1 into a single model.

## Results

We find similar results as in Study 1. Overconfidence is related positively toward EGM attitudes but is unrelated to SGM attitudes. We produce a similar effect size as in Study 1. As shown in Table 5, our estimates of the EGM overconfidence scale imply a 0.172–0.190 increase in attitude factor scores, for each unit increase in overconfidence. Consistent with prior literature that shows a limited effect of messaging, we find no statistically significant impact of the chance or skill labeling conditions. We also find that individuals in the low and moderate PGSI categories have more positive attitudes toward EGMs and SGMs. In this study, we found individuals in the low and problem categories had significantly higher attitudes toward EGMs than individuals in the non-problem category. However, we found individuals in problem category had significantly lower attitudes toward SGMs than non-problem gamblers.

**TABLE 5:** Ordinary least squares regression of overconfidence variables onto attitude factor variables (experiment).

	EGMs	EGMs	EGMs	SGMs	SGMs	SGMs
EGM-	0.190***	0.187***	0.172**			
Overconfidence	(0.041)	(0.043)	(0.053)			
SGM-				0.154	0.152	0.085
Overconfidence				(0.081)	(0.084)	(0.101)
Told chance		−0.012	−0.154		0.240	0.334
		(0.198)	(0.213)		(0.231)	(0.219)
Told skill		−0.083	−0.219		0.054	0.280
		(0.231)	(0.240)		(0.202)	(0.205)
PGSI low			0.579**			0.151
			(0.200)			(0.198)
PGSI moderate			0.444			0.118
			(0.326)			(0.303)
PGSI problem			0.729*			−0.876**
			(0.312)			(0.326)
Age			0.015			−0.018*
			(0.008)			(0.008)
Constant	−0.000	0.030	−0.518	0.000	−0.099	−0.938
	(0.085)	(0.150)	(0.556)	(0.089)	(0.162)	(0.742)
Gender	No	No	Yes	No	No	Yes
Employment	No	No	Yes	No	No	Yes
Household income	No	No	Yes	No	No	Yes
*N*	122	122	122	124	124	117
*R*^2^	0.116	0.117	0.443	0.029	0.040	0.372

*p < 0.05, **p < 0.01, ***p < 0.001. Heteroskedasticity robust standard errors shown in brackets. PGSI measures are binary variables representing categories in the problem gambling severity index, with non-problem/non-gambling excluded as the base category.

## Discussion

The advent of a new form of machine gambling, which involves a skill component, provided an opportunity to examine the role of game (mis)understanding in consumer attitudes toward EGMs. Our hypothesis was supported, as our results demonstrated that EGMs are systematically misunderstood by individuals, resulting in cognitive biases that relate strongly to attitudes. These findings highlight the importance of overconfidence as maladaptive thought that relates to positive affect toward EGMs. Results from both studies showed a non-normal distribution of overconfidence toward EGM understanding, and that as overconfidence increased, participant’s positive attitudes toward playing EGMs also increased. This finding was robust and did not change in relation to personal characteristics, level of problem gambling severity, or whether participants were provided with accurate or inaccurate information about how EGMs worked. The same relationship was not found for SGMs, which may be related to the absence of a relationship or to a much lower frequency of influential extreme values than was observed in the case of EGMs.

The provision of accurate and inaccurate information about how outcomes were determined did not influence attitudes. As positive attitudes have been shown to predict intent to play EGMs (Gainsbury et al., 2019a), individuals who are overconfident that they understand EGMs may be more likely to play the devices. This may lead to negative outcomes as previous research shows inaccurate understanding of EGMs and erroneous beliefs is related to gambling problems (Goodie et al., 2019). The current research suggests that the erroneous beliefs may influence behavior due to their influence on attitudes, which is consistent with the learning and cognitive pathway mentioned in several psych-social models of gambling behavior (e.g., Blaszczynski and Nower (2002). This finding did not hold for SGMs and participants had a more normal distribution of overconfidence values in their understanding of SGMs.

Despite the appearance of more extreme values of EGMs overconfidence, both studies showed participants had higher measured understanding and lower self-reported understanding of EGMs than SGMs. While this may relate to the distribution of the underlying measures, it may also suggest that there is a subset of individuals with a highly distorted view of their EGM understanding and warrants further study.

Based on our results, it may be expected that SGM players would be more effective at moderating their risk taking behaviors, based on their understanding or lack of certainty of how outcomes are determined, in comparison to EGMs. That is, relative to EGMs, SGM players recognize the limits of their understanding, and have attitudes that are uncorrelated with the degree of overconfidence. The findings may indicate that when faced with a novel activity, individuals have less well-developed biases. SGMs were entirely new to Study Two participants and Study One participants had limited experience with the devices.

Alternatively, the increased interactivity and complexity of SGMs may be less likely to lead to cognitive biases than randomly determined games, given human biases to look for patterns (Kahneman and Tversky, 2013). Social and cognitive psychology research suggests that when performing challenging tasks with a focus on acquiring an incremental skill that can be enhanced through effort, people focus on learning, use analytical strategies, and have high self-efficacy (Wood and Bandura, 1989). The greater perceived role of skill in SGMs may keep individuals focused on the activity and how their actions influence outcomes, with the constant feedback and shifting efforts reducing a sense of overconfidence developing that influences attitudes. We note that in Study 2, we observed that individuals in the PGSI problem gambling category had significantly higher attitudes toward EGMs than non-problem gamblers, but significantly lower attitudes toward SGMs. Given the lack of research on SGMs, the interpretation of these findings are hypothetical and warrant further research.

The research outcomes are somewhat surprising as EGMs should be easier to understand in terms of how outcomes are determined than SGMs, yet participants had higher rated understanding of SGMs. Outcomes are completely chance based for EGMs, with no influence of any external or personal factors. In contrast, SGMs include many different formats, skill plays a differing and inconsistent role, and chance is still the predominant factor but to an undefined extent. As such, it is rational that participants indicated uncertainty in their understanding of SGMs to a greater extent than EGMs. The findings suggest that this awareness of the lack of understanding may play a protective factor as it was not related to attitudes to play, which is related to intention in the literature. Previous research has found that individuals with greater gambling-related irrational beliefs are more likely to play SGMs, but that understanding did not influence intent to play (deidentified). Study Two supports this finding in a new sample and based on random allocation to exposure to SGMs.

Given previous findings that positive attitudes predict intention to play EGMs (Gainsbury et al., 2019a), our results are similar to Goodie (2005), who found that individuals with greater confidence in their understanding of how the outcomes are determined were more likely to engage in EGM gambling activity. Our findings suggest that decision-making underlying gambling behavior may be related to individual cognitive processes and decision strategies. Therefore, effective policies and practices to influence cognitive processes and decision-making capabilities could influence EGM gambling behavior. However, there was no impact of the labeling of machines as being based on either chance or skill, including when this information was accurate or inaccurate. This is consistent with previous research that messages providing information to inform EGM play is ineffective in altering cognitions or behaviors (Monaghan and Blaszczynski, 2010). The labeling may have been too subtle to influence attitudes given the highly impactful stimulus of SGMs and EGMs. The results support previous findings suggesting that messages need to attract attention to have any impact, such as being presented on machine screens during a break in play (Gainsbury et al., 2015; Landon et al., 2016; Ginley et al., 2017; Harris et al., 2018; Critchlow et al., 2020).

SGM understanding was higher among participants in Study One than in Study Two. Given that 43% of participants in Study One had some prior experience with SGMs, this may indicate that previous gambling opportunities shapes understanding, although Study Two participants also had a chance to play the SGM, albeit in a simulated environment. Given the differences between the participant groups, further research is needed to assess the impact of SGM play on consumer understanding of how outcomes are determined.

SGM understanding was not impacted by framing in the current research. The framing was relatively minor, but the results suggest that play experience or perception of machines, not how these are describes shapes attitudes. The lack of attention and comprehension of information provided about how outcomes are determined is consistent with research about informative messaging (Monaghan and Blaszczynski, 2009; Ginley et al., 2017) and may account for misunderstanding of EGMs. Although the findings are preliminary, the implications are that labeling needs to be much stronger and more persuasive to change beliefs and misconceptions. Further research is required regarding the optimal timing for messages to shape attitudes as messages may be more impactful after initial engagement given that messages prior to play had no impact, but these need to be strong enough to counter experience.

### Limitations and Future Research

The results from these studies need to be considered in terms of the methodological limitations of the research. Both samples were self-recruited and were non-probabilistic or representative of the broader population of consumers likely to play EGMs and SGMs. The results are based on self-report which may be biased and not accurately capture understanding of the machines or true attitudes. To reduce the potential bias in self-report responses we asked participants on feedback of both EGMs and SGMs and used the PGSI to control for prior gambling exposure and experience of harms. There was no gambling behavior measured in either study. Nonetheless, given the precautionary principle (O’Riordan and Cameron, 1994; Raffensperger and Tickner, 1999), it is important for research to examine SGMs in jurisdictions before these are considered for regulatory approval. The benefit of laboratory-based research enables random allocation to be exposed to EGMs or SGMs, which would not be possible in a real-world trial. Given the inherent limitations associated with laboratory-based research, we recommend real-world trials in controlled venues to investigate engagement amongst a broader cohort with SGMs including those familiar with gaming without gambling experience as well as those already highly engaged with other gambling activities to assess the impact on real gambling behavior and outcomes.

#### Implications

This study makes an important Contribution To The Field. It furthers the available literature regarding SGMs and builds on wider research demonstrating connections in understanding, cognitive biases, and attitudes between populations. These findings suggest that in contrast to a tendency for individuals to over-estimate their knowledge of how EGMs work, which leads to positive attitudes with a subsequent impact on decision-making and behavior, individuals are cautious in their accuracy of understanding SGMs and there is no observed biases which influence attitudes. This is an important outcome as we found no evidence to suggest that when exposed to SGMs, individuals will over-emphasize the role of skill and this will influence their attitudes toward SGMs and subsequent intent to play these machines. Even considering the limitations of the research, this is an important finding for regulators considering the impact of SGMs. Further research is needed to examine the impact of SGMs in a licensed gambling environment and to consider differences between cohorts in terms of overconfidence in understanding SGMs and the influence of this on attitudes and subsequent intention and behavior.

## Data Availability Statement

The datasets presented in this study can be found in online repositories. The names of the repository/repositories and accession number(s) can be found below: https://osf.io/ba5n2/.

## Ethics Statement

The studies involving human participants were reviewed and approved by the University of Sydney Human Research Ethics Committee. The patients/participants provided their written informed consent to participate in this study.

## Author Contributions

Both authors listed have made a substantial, direct and intellectual contribution to the work, and approved it for publication.

## Conflict of Interest

Funding for Study 2 was received from Wymac Gaming Solutions who also provided the machines used in the research. Wymac had no input into the design, methodology, research conduct, analysis, or publication process. Both authors maintain disclosure statements at: https://www.tandfonline.com/action/journalInformation?show=editorialBoard&journalCode=rigs20.

## Footnotes

1 Available upon reasonable request to the author.

2 We thank our editor for this insight.

3 For a demonstration of the specific SGM used please see https://www.youtube.com/watch?v=9ga7UC6zL_s
